# Pre-existing anti-HCoV-OC43 immunity influences the durability and cross-reactivity of humoral response to SARS-CoV-2 vaccination

**DOI:** 10.3389/fcimb.2022.978440

**Published:** 2022-09-02

**Authors:** Caiqin Hu, Zheng Wang, Li Ren, Yanling Hao, Meiling Zhu, He Jiang, Shuo Wang, Dan Li, Yiming Shao

**Affiliations:** ^1^ State Key Laboratory for Diagnosis and Treatment of Infectious Diseases, National Clinical Research Center for Infectious Diseases, National Medical Center for Infectious Diseases, Collaborative Innovation Center for Diagnosis and Treatment of Infectious Diseases, The First Affiliated Hospital, Zhejiang University School of Medicine, Zhejiang, China; ^2^ State Key Laboratory for Infectious Disease Prevention and Control, National Center for AIDS/STD Control and Prevention, Chinese Center for Disease Control and Prevention, Beijing, China; ^3^ Guangxi Key Laboratory of AIDS Prevention and Control and Achievement Transformation, Guangxi Center for Disease Prevention and Control, Nanning, China

**Keywords:** SARS-CoV-2, seasonal human coronavirus, antibody, cross-reactivity, protective immunity

## Abstract

**Purpose:**

This study was conducted in order to properly understand whether prior seasonal human coronavirus (HCoV) immunity could impact the potential cross-reactivity of humoral responses induced by SARS-CoV-2 vaccine, thereby devising universal coronavirus vaccines for future outbreaks.

**Methods:**

We performed enzyme-linked immunosorbent assay (ELISA) to quantify the immunoglobulin G (IgG) antibody levels to spike (S) protein and S1 subunit of HCoVs (HCoV-OC43, HCoV-HKU1, HCoV-NL63, and HCoV-229E), and ELISA [anti-RBD and anti-nucleoprotein (N)], chemiluminescence immunoassay assays (anti-RBD), pseudovirus neutralization test, and authentic viral neutralization test to detect the binding and neutralizing antibodies to SARS-CoV-2 in the vaccinees.

**Results:**

We found that the antibody of seasonal HCoVs did exist before vaccination and could be boosted by SARS-CoV-2 vaccine. A further analysis demonstrated that the prior S and S1 IgG antibodies of HCoV-OC43 were positively correlated with anti-RBD and neutralization antibodies to SARS-CoV-2 at 12 and 24 weeks after the second vaccination, and the correlation is more statistically significant at 24 weeks. The persistent antibody levels of SARS-CoV-2 were observed in vaccinees with higher pre-existing HCoV-OC43 antibodies.

**Conclusion:**

Our data indicate that inactivated SARS-CoV-2 vaccination may confer cross-protection against seasonal coronaviruses in most individuals, and more importantly, the pre-existing HCoV-OC43 antibody was associated with protective immunity to SARS-CoV-2, supporting the development of a pan-coronavirus vaccine.

## Introduction

Since the COVID-19 pandemic status was declared by WHO on March 11, 2020, the prevalence of SARS-CoV-2 has been a major concern for clinicians and researchers. In addition to SARS-CoV-2, six other coronaviruses (SARS-CoV, MERS, HCoV-OC43, HCoV-HKU1, HCoV-NL63, and HCoV-229E) could also infect humans (Su et al., 2016; Corman et al., 2018). Patients of SARS-CoV, MERS, and SARS-CoV-2 have a variety of clinical manifestations, ranging from mild to severe and even death. While the endemic and seasonal HCoVs, including HCoV-OC43, HCoV-HKU1, HCoV-NL63, and HCoV-229E, typically cause upper respiratory symptoms and are usually self-limited in human. Unlike SARS-CoV and MERS, the seroprevalence could reach more than 90% for at least three of the seasonal HCoVs and account for at least 30% of seasonal colds (Severance et al., 2008; Gorse et al., 2010; Su et al., 2016; Chen et al., 2020; Jin et al., 2020).

SARS-CoV-2 vaccines could limit symptom burden, cutting down the number of hospitalizations and deaths (Al Kaabi et al., 2021; Bergwerk et al., 2021; Khoury et al., 2021), but reducing the efficacy against newly emerging variants and increasing cases of breakthrough infection had continuously been reported (Edara et al., 2021; Zhou et al., 2021; Muik et al., 2022). Knowledge of SARS-CoV-2 immunity, especially the cross-reactivity of antibodies against different coronaviruses, is critical for understanding the potential future immunity of vaccinees to other HCoVs and providing guidance for pan-coronavirus vaccine development.

In this study, we focused on the serologic antibodies of seasonal HCoVs and SARS-CoV-2 within 6 months post-vaccination and characterized the relationship among these coronaviruses. We performed enzyme-linked immunosorbent assay (ELISA) to test the immunoglobulin G (IgG) antibody levels to spike (S) protein and S1 subunit of HCoVs and ELISA [anti-RBD and anti-nucleoprotein (N)], chemiluminescence immunoassay assays (CLIA, anti-RBD), pseudovirus neutralization test (PVNT), and authentic viral neutralization test to detect the binding and neutralizing antibodies to SARS-CoV-2 in the vaccinees.

## Materials and methods

### Study participants

A total of 23 healthy people who have completed two doses of BBIBP-CorV vaccination were enrolled, and their informed consent was obtained. The study was conducted in accordance with the Declaration of Helsinki, and the protocol was approved by the Ethics Committee of the First Affiliated Hospital, Zhejiang University School of Medicine (reference number 2021376).

### WANTAI SARS-CoV-2 Ab enzyme-linked immunosorbent assay

The anti-RBD and anti-N antibodies of SARS-CoV-2 were measured using SARS-CoV-2 RBD/N ELISA kits, respectively, in accordance with the instructions (Wantai Biological Pharmacy, China). Briefly, 96-well plates were pre-coated with 2019-nCoV-RBD antigen (recombinant protein expressed in HEK 293 T cells) or 2019-nCoV-N antigen (recombinant protein expressed in *E*. *coli*). Furthermore, 100 µl of plasma samples was added, incubated for 0.5 h at 37°C, and washed. Then, 100 µl of horseradish peroxidase (HRP)-labeled 2019-nCoV-Ag was incubated for 0.5 h at 37°C and washed. After that, 100 µl of substrate was added in each well for 15 min at 37°C. The reaction was stopped by adding 50 µl of 1 nm H_2_SO4 to each well, and the reading was taken at 450 nm. The amino acid sequences of 2019-nCoV-RBD and 2019-nCoV-N are presented in **Supplementary Table S3**.

### Enzyme-linked immunosorbent assay of seasonal human coronavirus

The spike protein and S1 subunits of seasonal hCoVs (HCoV-OC43, HCoV-HKU1, HCoV-NL63, and HCoV-229E) were purchased from Sino Biological (catalog numbers 40607-V08B, 40607-V08H, 40606-V08B, 40602-V08H, 40604-V08B, 40600-V08H, 40605-V08B, and 40601-V08H, respectively). Furthermore, 100 µl of antigens at a concentration of 0.5 ug/ml was coated in 96-well ELISA plates overnight at 4°C. The plate was washed with PBS-T (0.05% Tween 20) five times and blocked with 250 ul of blocking buffer (PBS, ph 7.4, + 2% BSA + 5%milk) for 2 h at 37°C. The blocking solution was removed, and diluted serum samples from a starting concentration of 1:100 were incubated at 37°C for 1 h. The plates were washed five times with PBS-T and 100 μl HRP-goat anti-human IgG (1:5,000 dilution) in diluted blocking buffer for 1 h at 37°C. The plates were washed with PBST five times. Then, 100 ul of substrate was added in each well for 15 min at room temperature. The reaction was stopped by adding 50 µl of 1 nm H_2_SO4 to each well, and the reading was taken at 450 nm.

### Chemiluminescent microparticle immunoassay

The COVID-19 nAbs detection kits (Hotgen, Beijing, China, batch number: 21010115) were based on chemiluminescent microparticle immunoassay and used a competitive ELISA method to detect SARS-CoV-2 nAbs. In short, the test was performed by reacting the sample with alkaline phosphatase-labeled S-RBD antigen complex. Biotin-labeled receptor protein ACE2 and magnetic microspheres encapsulated with streptavidin were added to promote the attachment of the ACE2-S-RBD antigen complex to the magnetic microspheres by the specific binding of biotin and streptavidin. A matched automatic chemiluminescence immunoassay analyzer was used to analyze the nAb levels which were presented as the chemiluminescence signal values divided by the cutoff (absorbance/cutoff, S/CO). All operations were carried out in strict accordance with the instructions of the reagent manufacturer. Furthermore, 100 μl plasma sample was added, and the whole process took about 30 min. S/CO <1 was considered positive, while S/CO ≥1 was considered negative.

### Pseudovirus neutralization test

The pseudovirus was generated by co-transfection of HEK 293 T cells with pcDNA3.1-S-COVID19 and pNL4-3Luc, which carries the optimized spike (S) gene and a human immunodeficiency virus type 1 backbone, respectively. Then, 150-ul serial dilutions of human sera (four serial threefold dilutions in Dulbecco’s minimum essential medium with an initial dilution of 1:20) were added into 96-well plates. After that, 50 ul pseudoviruses of SARS-CoV-2 with a concentration of 1,300 TCID50/ml was added into the plates, followed by incubation at 37°C for 1 h. Afterward, Hu-h7 cells were added into the plates (1.5 × 10^4^ cells/100 ul cells per well), followed by incubation at 37°C in a humidified atmosphere with 5% CO_2_. Chemiluminescence detection was performed after 48 h of incubation. The Reed–Muench method was used to calculate the virus neutralization titers. The result was reported as half-maximal inhibitory concentration of PVNT (PVNT50).

### Live viral neutralization test

The live viral neutralization test of SARS-CoV-2 was performed as previously described (Li et al., 2022). In brief, the neutralizing antibody titers against the wild-type strain and the variants (Beta B.1.1.7, Gamma P.1, and Delta B.1.617) in serum were determined by using a cytopathic effect-based microneutralization assay in Vero cells (National Collection of Authenticated Cell Cultures, National Academy of Science, China). The serum was then mixed with the same volume of viral solution to achieve a final concentration of 100 TCID50 per well. The reported titer was the reciprocal of the highest sample dilution that protected at least 50% of the cells from cytopathic effects. Serum dilution for the neutralization assay started from 1:4, and seropositivity was defined as titer ≥1:4.

### Multiple sequence alignment

Multiple sequence alignment (MSA) that used to determine the spike protein and nucleoprotein sequence’s identity and similarity among SARS-CoV-2 (MN908947.3), the seasonal human coronaviruses HKU1 (DQ415914.1), OC43 (MF374985), 229E (NC002645.1), and NL63 (AY567487.2) were aligned and analyzed by the BLOSUM50 method of MatGAT (version 2.0). The conservation and antigenic epitopes of SARS-CoV-2 RBD protein were conducted by the JaIview (version 2.11) and EMBOSS explorer (https://www.bioinformatics.nl/cgi-bin/emboss/antigenic), respectively.

### Statistical analysis

All IgG antibody titers to seasonal HCoVs were log10-transformed to improve the linearity. The geometric mean titers (GMT) and 95% confidence intervals (95% CI) were computed as log10‐transformed titers. Two-tailed, nonparametric Mann–Whitney *U*-test and Kruskal–Wallis test were performed on numerical data. Pearson and linear correlation were used on antibody response against seasonal HCoVs and SARS-CoV-2. The graphs and statistical analyses were conducted using GraphPad Prism (version 8.0.2), Origin2021b (version 9.8.5), and SPSS software (version 23.0). *P*-values less than 0.05 were considered statistically significant.

## Results

### Sequence homology between SARS-CoV-2 and seasonal human coronaviruses

**Figure 1:**
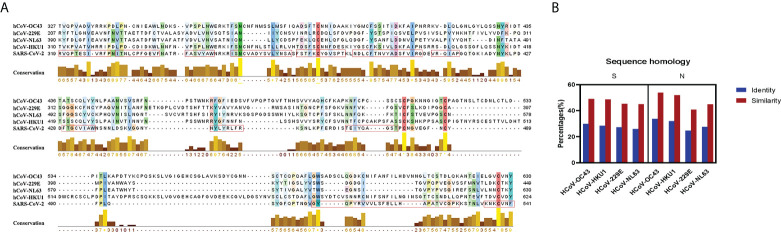
Sequence homology and conservation between SARS-CoV-2 and seasonal HCoVs. **(A)** The conservation and the antigenic epitopes of RBD are shown as a yellow bar chart and a red rectangle. The amino acid conservation scores were classified into 10 levels (1, most variable; +, most conserved). **(B)** Sequence homology and conservation of spike protein and nucleoprotein between SARS-CoV-2 and seasonal HCoVs. The identity and the similarity (%) of these two proteins were aligned and analyzed by the BLOSUM50 method of MatGAT (version 2.0) (James and Campanella*, 2003).

To better evaluate the potential cross-reactivity, we compared the amino acid sequence of spike protein and nucleoprotein among SARS-CoV-2, HCoV-OC43, HCoV-HKU1, HCoV-NL63, and HCoV-229E. We also estimated the relative conservation scores and antigenic epitopes of the RBD region of SARS-CoV-2. The conservation and antigenic epitopes were conducted by the JaIview and EMBOSS explorer, respectively (**Figure 1A**). MSA was analyzed by the BLOSUM50 method of MatGAT (version 2.0) (James and Campanella*, 2003). The greatest homology of spike was between SARS-CoV-2 and HCoV-OC43 (29.8% identity and 49% similarity), followed by HCoV-HKU1(28.5% identity and 48.6% similarity), HCoV-229E (27.3% identity and 45.2% similarity), and HCoV-NL63 (25.9% identity and 45% similarity). The greatest homology of nucleoprotein was between SARS-CoV-2 and HCoV-OC43 (33.7% identity and 53.8% similarity), followed by HCoV-HKU1(31.9% identity and 51.9% similarity), HCoV-NL63 (27.6% identity and 44.9% similarity), and HCoV-229E (24.7% identity and 40.8% similarity) (**Figure 1B**). Both S and N proteins of HCoV-OC43 and HCoV-HKU1 were closer to SARS-CoV-2 than those of HCoV-NL63 and HCoV-229E.

### Cross-reactivity of antibodies boosted by SARS-CoV-2 vaccine

**Figure 2:**
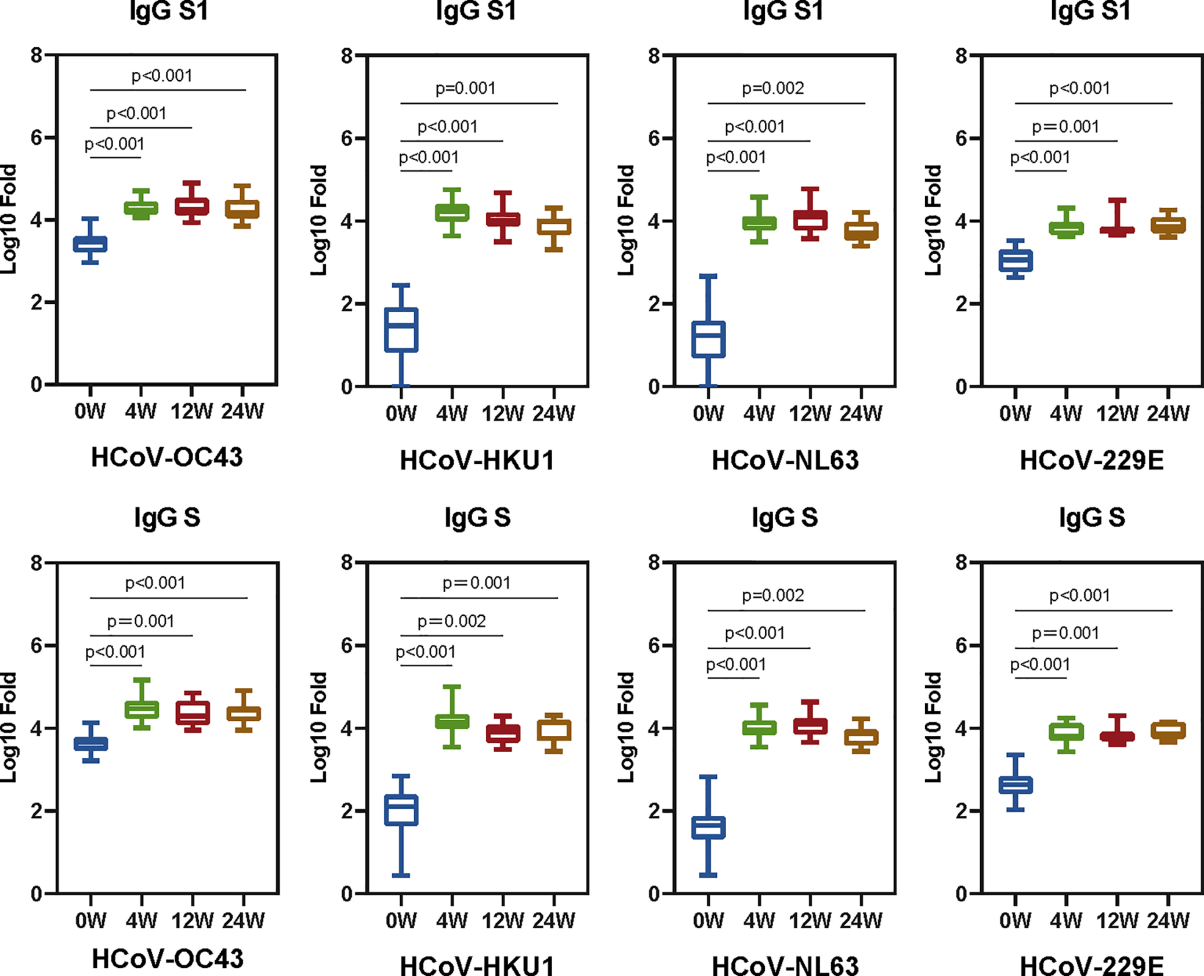
The SARS-CoV-2 vaccine boosts antibodies that are reactive to seasonal HCoVs. We quantified the serum IgG antibodies to the S and S1 proteins against HCoVs (HCoV-OC43, HCoV-HKU1, HCoV-NL63, and HCoV-229E). The IgG geometric mean titers (GMT) against seasonal HCoVs spike and S1 are shown in the boxplot. Two-tailed, nonparametric Dunn’s Kruskal–Wallis test was used for multiple comparisons. The middle bars indicate the GMT values, the box indicates interquartile range, and the lines indicate minimum and maximum. Statistically significant values are marked in the figure.

To explore the antibody response induced by SARS-CoV-2 vaccine, 23 healthy people [17 female and six male, with a median age of 27 (interquartile range: 26–39) years old] who completed two doses of BBIBP-CorV vaccination were included in this study. The baseline characteristics of the healthy donors are presented in **Supplementary Table S1**. The interval between two doses of vaccine is 21 days. Plasma samples before the first dose of vaccination and at 4, 12, and 24 weeks after the second dose of inactivated BBIBP-CorV vaccine were collected to measure the antibody activity against seasonal HCoVs and the SARS-CoV-2. No neutralization and binding ability of SARS-CoV-2 were shown before vaccination (Hu et al., 2022). The IgG levels to spikes and S1 subunits of seasonal HCoVs were quantified as area under the curve (AUC) by plotting normalized optical density values against the serum sample dilutions for ELISAs (**Supplementary Figure S1**). The GMT (95% CI) at each time point for AUC ELISA against the seasonal HCoVs are shown in **Supplementary Table S2**. The serum IgG antibodies to the seasonal HCoVs pre-existed before the SARS-CoV-2 vaccine and were boosted after vaccination. Significant differences between groups are displayed (**Figure 2**).

### Correlation of antibody levels between SARS-CoV-2 with seasonal HCoVs

**Figure 3:**
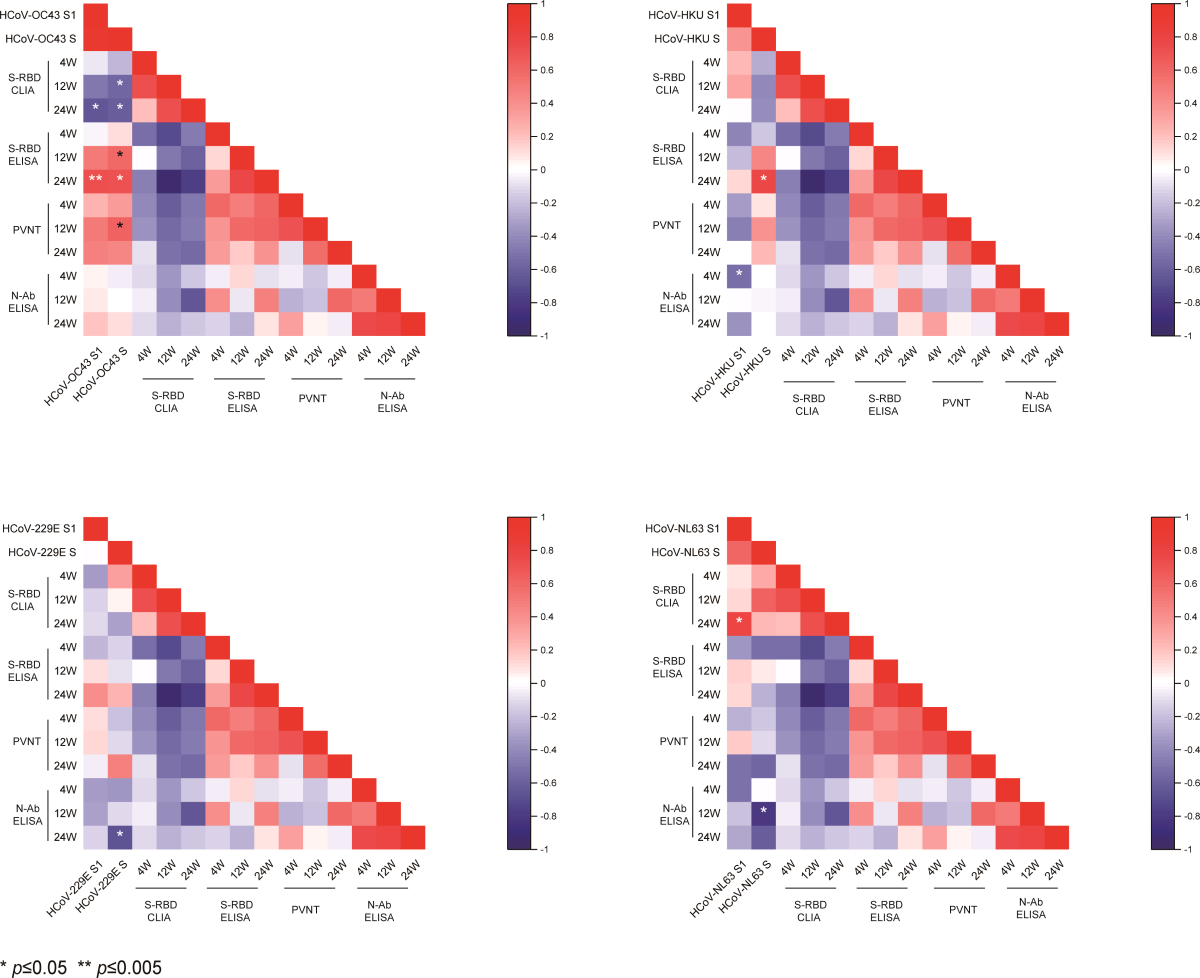
Pearson correlation matrices of antibody levels to SARS-CoV-2 and seasonal HCoVs. Heat map of the Pearson correlation matrices of pre-existing spike and S1 subunits’ IgG antibody levels to seasonal HCoVs (HCoV-OC43, HCoV-HKU1, HCoV-NL63, and HCoV-229E) and S-RBD/N antibody and nAbs to SARS-CoV-2 at 4, 12, and 24 weeks after vaccination. Statistically significant correlations are indicated with an asterisk (*) in the first two columns of every heat map. *P < 0.05, **P < 0.005.

**Figure 4:**
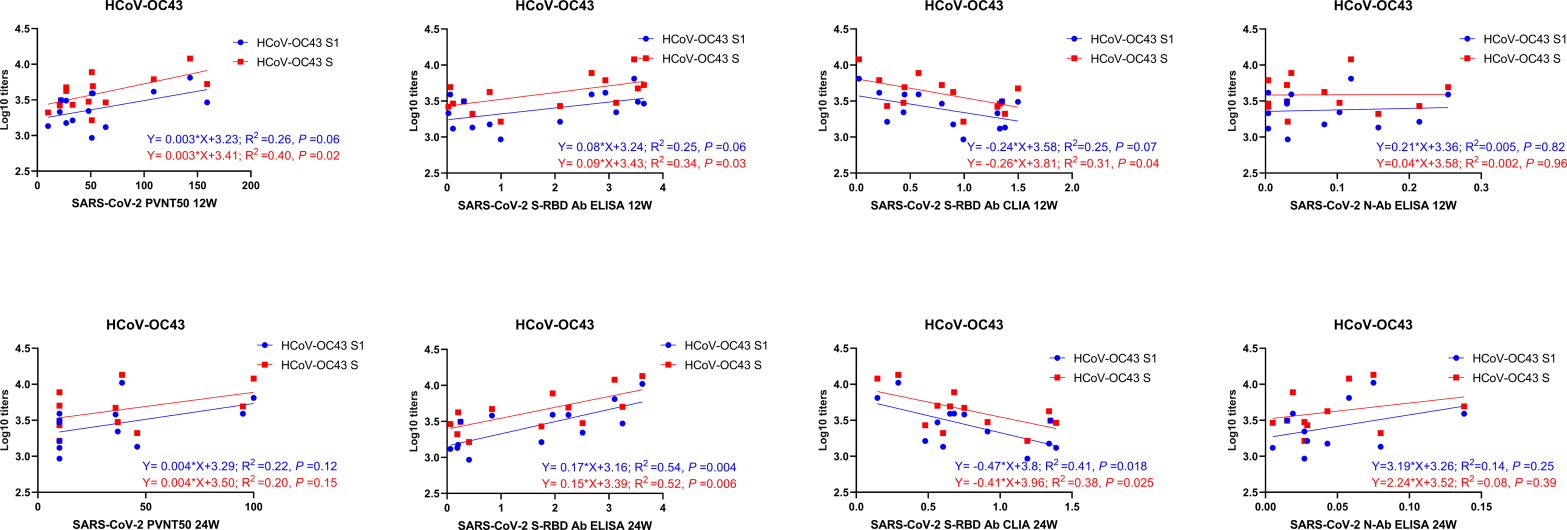
Linear correlation of antibody levels between SARS-CoV-2 and HCoV-OC43. The linear correlation between prior S/S1-IgG antibody levels to HCoV-OC43 and S-RBD/N/neutralizing antibodies to SARS-CoV-2 at 12 and 24 weeks after vaccination is shown in the figure. A scatter point represents a record. *R* ^2^ represents the degree of linear deviation obtained by fitting the experimental data. The *p*-value tests whether the regression equation is significant. The larger the *R* ^2^ value, the smaller the *p*-value. When the *p*-value is less than 0.05, this linear model can be considered valuable.

We analyzed the correlations of prior IgG levels against seasonal HCoVs and the RBD, N, and neutralizing antibodies of SARS-CoV-2 at 4, 12, and 24 weeks after vaccination. Pearson correlation matrices according to seasonal HCoV subtype are shown in **Figure 3**. At 12 weeks after vaccination, the prior S-IgG antibodies of HCoV-OC43 were positively correlated with S-RBD antibodies by ELISA and nAb and negatively correlated with S-RBD antibodies by CLIA to SARS-CoV-2, while the S1 IgG antibody of HCoV-OC43 was not correlated with SARS-CoV-2. At 24 weeks after vaccination, the prior S and S1 IgG antibodies to HCoV-OC43 were all shown to be related with the S-RBD antibodies to SARS-CoV-2. The S-RBD antibody by CLIA was detected with a competitive method; the lower values of CLIA mean a higher antibody activity. Similarly, it is also evidenced that the pre-existing antibodies’ response to other seasonal HCoVs (including HCoV-HKU1, HCoV-NL63, and HCoV-229E) were not related well with the humoral immunity to SARS-CoV-2. To show the relationship between the pre-existing antibody response to seasonal HCoVs and the S-RBD/N antibody and nAb to SARS-CoV-2 more clearly, the *R* ^2^ and *P*-values of the linear regressions are shown in **Figure 4**. The same results are shown in **Figure 3**; the correlations between prior S-IgG antibodies of HCoV-OC43 and S-RBD antibodies to SARS-CoV-2 at 24 weeks were stronger than at 12 weeks after the SARS-CoV-2 vaccine. In short, the higher the pre-existing antibody levels of HCoV-OC43, the higher the S-RBD binding antibodies against SARS-CoV-2, and the correlations tend to become stronger over time after vaccination.

### Higher prior IgG antibody levels to HCoV-OC43 predict durable antibody responses to SARS-CoV-2

**Figure 5:**
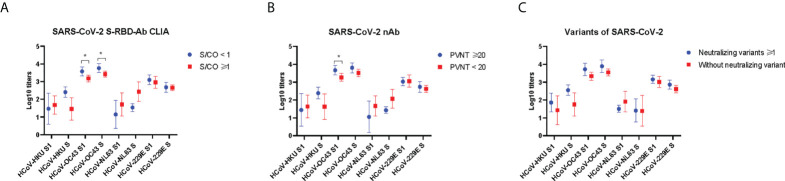
Prior antibody to seasonal HCoVs in the groups with and without antibodies to SARS-CoV-2 at 24 weeks after vaccination. The anti-RBD antibody levels were detected by chemiluminescent microparticle immunoassay **(A)**. The nAb levels were detected by pseudovirus neutralization test **(B)**, and neutralizing antibodies against authentic SARS-CoV-2 variants **(C)**. Two-tailed, nonparametric Mann–Whitney test was used for comparisons. The bars display the mean and SD. Part of the results between groups is shown in the figure. *p < 0.05.

As mentioned above, the correlations between prior S-IgG antibodies of HCoV-OC43 and S-RBD antibodies to SARS-CoV-2 were highest at 24 weeks post-vaccination. Moreover, the antibody responses to SARS-CoV-2 at 24 weeks after vaccination can be regarded as a long-term immune response. We then divided the vaccinees into the two groups, with or without antibody responses to SARS-CoV-2 at 24 weeks after vaccination. PVNT50 above 20 was considered to be with a neutralizing activity and that less than 20 as without a neutralizing activity to SARS-CoV-2. S/CO less than 1 was regarded with anti-RBD antibody and that above 1 as without anti-RBD antibody to SARS-CoV-2. The pre-existing IgG antibody to spike and S1 of HCoV-HKU1, HCoV-NL63, and HCoV-229E all showed no difference in neutralizing and anti-RBD antibodies to SARS-CoV-2 in the two groups. However, a higher prior level of S/S1-IgG antibodies was to HCoV-OC43 exhibited in the group with neutralizing or anti-RBD antibodies to SARS-CoV-2 than another group (**Figure 5**). It has been speculated that the pre-existing antibody to HCoV-OC43 was associated with protective immunity to SARS-CoV-2.

### Prior IgG antibody levels to seasonal HCoVs cannot influence the antibody activity to SARS-CoV-2 variants

Finally, in order to test whether prior HCoVs influence antibody responses against SARS-CoV-2 variants at 24 weeks after vaccination, we measured the levels of neutralizing antibodies against authentic SARS-CoV-2 variants, including Beta (B.1.1.7), Gamma (P.1), and Delta (B.1.617). Then, we divided the cohort into two groups: one group was able to neutralize at least one of the variants, and another was unable to neutralize the variants. Plasma dilution above 4 was considered to be with a neutralizing antibody and that less than 4 as without a neutralizing activity to SARS-CoV-2 variants. The pre-existing IgG antibody of seasonal HCoVs all showed no difference in the two groups (**Figure 5C**).

## Discussion

Two doses of BBIBP-CorV vaccines showed 100% seroconversion rate and 78.1% efficacy against symptomatic illness separately (Al Kaabi et al., 2021; Jeewandara et al., 2021). Even though China has achieved 88% coverage with SARS-CoV-2 full vaccination, there are still breakthrough infection cases reported in populations. In our previous study, we found that the binding and neutralizing antibody to SARS-CoV-2 reached peaks at 4 weeks after the second dose vaccination and then gradually declined over time (Hu et al., 2022).

Cross-reactivity immunity is necessary to properly interpret results from serologic studies. In our study, the levels of prior IgG antibody to HCoV-229E and HCoV-OC43 were higher than the levels of IgG antibody to HCoV-NL63 and HCoV-HKU1, which were similar to the data reported previously by Gorse GJ et al. (Gorse et al., 2010). Most individuals possessed pre-existing serum antibodies reactive to HCoV-OC43, HCoV-HKU1, HCoV-NL63, and HCoV-229E, and the SARS-CoV-2 vaccine can boost the the level of antibodies of seasonal HCoVs. These results were consistent with previous studies (Severance et al., 2008; Aguilar-Bretones et al., 2021; Anderson et al., 2021; Camerini et al., 2021; Hicks et al., 2021; Majdoubi et al., 2021). Guo L et al. also tested the antigenic cross-reactivities of S protein between SARS-CoV-2 and seasonal HCoVs, and a two-way cross-reactivity was identified between SARS-CoV-2 and HCoVs (Guo et al., 2021). Regions within S antigens with high antigenic similarity between HCoVs are potential targets of cross-reactive antibodies (XiaoYan Che et al., 2005; Tso et al., 2021). The cross-reactive anti-spike IgG antibodies target not only the S2 domain but also the S1 domain, as suggested by Shrwani K et al. (Shrwani et al., 2021).

We found that the higher levels of pre-existing S and S1 IgG antibodies to HCoV-OC43 before vaccination were correlated with anti-RBD binding and neutralizing antibodies to SARS-CoV-2 at 12 and 24 weeks post-vaccination, and the correlation is more statistically significant at 24 weeks. It might give a signal to a more durable humoral response to SARS-CoV-2 infection in the population with a higher level of pre-existing antibodies to HCoV-OC43. Antibodies with a neutralizing activity are considered quite important in SARS-CoV-2 protection (Hall et al., 2021; Khoury et al., 2021). We speculate that the pre-existing antibody to HCoV-OC43 was also associated with the protective immunity to SARS-CoV-2. This conclusion is controversial—a study showed that pre-existing HCoV cross-reactive antibodies were not associated with protection from SARS-CoV-2 infections (Anderson et al., 2021).The IgG of HCoVs was shown to have a higher significance in asymptomatic than symptomatic seropositive individuals. The authors have considered that the pre-existing cross-reactive HCoV antibodies may have a protective effect against SARS-CoV-2 infection and the severity of COVID-19 disease (Ortega et al., 2021). The inhibitor of HCoV-OC43 also exhibited broad fusion inhibitory activity against multiple HCoVs, including SARS-CoV-2 (Xia et al., 2019).

The humoral immunity discussed in this article is also complementary to previous literatures on cellular immunity. Some cell immunity studies have been carried out on SARS-CoV-2 and HCoVs. Aguilar-Bretones M et al. had elucidated the frequency of HCoV-OC43-S1-specific B cells that showed a positive correlation with PRNT50 titers (Aguilar-Bretones et al., 2021). Loyal L et al. had shown that the pre-existing cross-reactive CD4^+^ T cells enhance the immune responses in SARS-CoV-2 infection and BNT162b2 vaccination (Loyal et al., 2021). Kundu R et al. put the conclusion straightly that high levels of pre-existing T cells of seasonal HCoVs can protect against SARS-CoV-2 infection (Kundu et al., 2022). These conclusions of cellular immunity further support our results. The pre-existing antibody to HCoV-OC43 may provide protective immunity to SARS-CoV-2.

Our study has several limitations. First, although we conducted a 24-week follow-up on vaccinees, the number of cases were relatively small. Second, low-pathogenic seasonal HCoVs typically cause self-limited colds with mild upper respiratory symptoms, so pathogenic detection is not routinely performed. Therefore, retrospective information on coronavirus infection cannot be effectively obtained. Third, the article was focused on humoral immunity in the vaccinated populations. The mechanism of the diversity in antibody responses after vaccination should be further explored in our study.

In conclusion, our study implied that the humoral immunity of seasonal HCoVs can be considered an important factor in assessing the antibody response to SARS-CoV-2. The results also suggest that the significant cross-reactive immune recognition between seasonal HCoVs and SARS-CoV-2 and the pre-existing HCoV-OC43 antibodies can prevent from SARS-CoV-2 infection. These findings may altogether shed new light on future vaccine strategies targeting newly emerging variants.

## Data availability statement

The original contributions presented in the study are included in the article/**Supplementary Material**. Further inquiries can be directed to the corresponding authors.

## Ethics statement

This study was granted by the Ethics Committee of First Affiliated Hospital, Zhejiang University School of Medicine (no. 2021376). The patients/participants provided their written informed consent to participate in this study.

## Author contributions

Conceptualization: CH, DL, and YS. Funding acquisition: ZW and YS. Methodology: CH, ZW, LR, YH, MZ, SW, and HJ. Resources: DL and YS. Software: CH, HJ, and DL. Writing—original draft: CH. Writing—review and editing: DL and YS. All authors contributed to the article and approved the submitted version.

## Funding

This research was funded by the National Natural Science Foundation of China under grant no. 20A20362 and Beijing Municipal Natural Science Foundation (M21015), the State Key Laboratory of Infections Disease Prevention and Control under grant no. 2020SKLID304 and no. 2020SKLID102, and the Science and Technology Project of Beijing under grant no. Z211100002521024.

## Acknowledgments

The authors thank all study participants.

## Conflict of interest

The authors declare that the research was conducted in the absence of any commercial or financial relationships that could be construed as a potential conflict of interest.

## Publisher’s note

All claims expressed in this article are solely those of the authors and do not necessarily represent those of their affiliated organizations, or those of the publisher, the editors and the reviewers. Any product that may be evaluated in this article, or claim that may be made by its manufacturer, is not guaranteed or endorsed by the publisher.
