# Supplementary material for: Pre-existing anti-HCoV-OC43 immunity influences the durability and cross-reactivity of humoral response to SARS-CoV-2 vaccination
Source: Front Cell Infect Microbiol. 2022 Sep 2;12:978440. doi: 10.3389/fcimb.2022.978440 (PMC9478943; doi:10.3389/fcimb.2022.978440)
Supplement: Supplementary file 1 [file DataSheet_1.docx]

**Supplementary Information: Pre-existing anti-HCoV-OC43 immunity influences the durability and cross-reactivity of humoral response to SARS-CoV-2 vaccination**

**Figure S1.** Binding profile of the serial dilution curve of serum samples collected at baseline, 4 weeks, 12 weeks and 24 weeks after vaccination. ELISAs assays were performed for the seasonal HCoVs antigens: IgG spike and S1. IgG levels of seasonal HCoVs were quantified as area under the curve (AUC) by plotting normalized optical density (OD) values.

**Table S1** Baseline characteristics of healthy donors

| Healthy donors | | | | | |
| --- | --- | --- | --- | --- | --- |
| ID | Gender | Age (years) | ID | Gender | Age (years) |
| HD01 | Female | 32 | HD13 | Female | 39 |
| HD02 | Female | 26 | HD14 | Female | 26 |
| HD03 | Female | 30 | HD15 | Male | 24 |
| HD04 | Female | 26 | HD16 | Female | 27 |
| HD05 | Female | 46 | HD17 | Female | 27 |
| HD06 | Female | 30 | HD18 | Female | 27 |
| HD07 | Female | 37 | HD19 | Female | 27 |
| HD08 | Male | 40 | HD20 | Female | 25 |
| HD09 | Female | 27 | HD21 | Male | 38 |
| HD10 | Female | 53 | HD22 | Male | 39 |
| HD11 | Male | 25 | HD23 | Male | 24 |
| HD12 | Female | 48 |  |  |  |

**Table S2** Antibody responses to seasonal human coronaviruses

|  | Before vaccination | 4 weeks after vaccination | 12 weeks after vaccination | 24 weeks after vaccination |
| --- | --- | --- | --- | --- |
| HCoV-HKU1 IgG S1 | 121（12-1206） | 13490（2407-75614） | 22223（4296-114921） | 11926（9572-14859） |
| HCoV-HKU1 IgG S | 328（63-1697） | 14866（9086-24322） | 10527（6806-18910） | 9952（2791-35481） |
| HCoV-OC43 IgG S1 | 5015（997-25241） | 24429（6436-92726） | 32787（4703-228560） | 23458（2442-225320） |
| HCoV-OC43 IgG S | 6819（1558-29840） | 42413（16360-109977） | 35473（7027-179102） | 30402（3530-261879） |
| HCoV-NL63 IgG S1 | 26（1-32433） | 8037（1064-60716） | 15101（620-367536） | 10762（3933-29451） |
| HCoV-NL63 IgG S | 33（5-234） | 7682（1230-47973） | 15693（1353-181970） | 12162（4318-34260） |
| HCoV-229E IgG S1 | 1292（444-3761） | 8408（6118-11556） | 9897（805-121730） | 9961（2650-37446） |
| HCoV-229E IgG S | 723（497-1050） | 11005（7024-17242） | 8676（1399-53790） | 8449（2762-25840） |

GMT: geometric mean titer; CI 95%: confidence interval 95%; IgG: immunoglobulin G; S: spike;

**Table S3** Amino acids sequence of 2019-nCoV-RBD and 2019-nCoV-N

| Protein | Amino acids sequence |
| --- | --- |
| 2019-nCoV-RBD | RVQPTESIVRFPNITNLCPFGEVFNATRFASVYAWNRKRISNCVADYSVLYNSASFSTFKCYGVSPTKLNDLCFTNVYADSFVIRGDEVRQIAPGQTGKIADYNYKLPDDFTGCVIAWNSNNLDSKVGGNYNYLYRLFRKSNLKPFERDISTEIYQAGSTPCNGVEGFNCYFPLQSYGFQPTNGVGYQPYRVVVLSFELLHAPATVCGPKKSTNLVKNKCVNFHHHHHH |
| 2019-nCoV-N | MSDNGPQNQRNAPRITFGGPSDSTGSNQNGERSGARSKQRRPQGLPNNTASWFTALTQHGKEDLKFPRGQGVPINTNSSPDDQIGYYRRATRRIRGGDGKMKDLSPRWYFYYLGTGPEAGLPYGANKDGIIWVATEGALNTPKDHIGTRNPANNAAIVLQLPQGTTLPKGFYAEGSRGGSQASSRSSSRSRNSSRNSTPGSSRGTSPARMAGNGGDAALALLLLDRLNQLESKMSGKGQQQQGQTVTKKSAAEASKKPRQKRTATKAYNVTQAFGRRGPEQTQGNFGDQELIRQGTDYKHWPQIAQFAPSASAFFGMSRIGMEVTPSGTWLTYTGAIKLDDKDPNFKDQVILLNKHIDAYKTFPPTEPKKDKKKKADETQALPQRQKKQQTVTLLPAADLDDFSKQLQQSMSSADSTQA |
